# Disparities in Mistreatment During Childbirth

**DOI:** 10.1001/jamanetworkopen.2024.4873

**Published:** 2024-04-04

**Authors:** Chen Liu, Kristen Underhill, Janice J. Aubey, Goleen Samari, Heidi L. Allen, Jamie R. Daw

**Affiliations:** 1 Department of Health Policy and Management, Columbia University Mailman School of Public Health, New York, New York; 2 Cornell Law School, Ithaca, New York; 3 Department of Obstetrics and Gynecology, NewYork-Presbyterian/Columbia University Medical Center, New York, New York; 4 Department of Population and Public Health Sciences, Keck School of Medicine, University of Southern California, Los Angeles; 5 Columbia University School of Social Work, New York, New York

## Abstract

**Importance:**

Lack of respectful maternity care may be a key factor associated with disparities in maternal health. However, mistreatment during childbirth has not been widely documented in the US.

**Objectives:**

To estimate the prevalence of mistreatment by health care professionals during childbirth among a representative multistate sample and to identify patient characteristics associated with mistreatment experiences.

**Design, Setting, and Participants:**

This cross-sectional study used representative survey data collected from respondents to the 2020 Pregnancy Risk and Monitoring System in 6 states and New York City who had a live birth in 2020 and participated in the Postpartum Assessment of Health Survey at 12 to 14 months’ post partum. Data were collected from January 1, 2021, to March 31, 2022.

**Exposures:**

Demographic, social, clinical, and birth characteristics that have been associated with patients’ health care experiences.

**Main Outcomes and Measures:**

Any mistreatment during childbirth, as measured by the Mistreatment by Care Providers in Childbirth scale, a validated measure of self-reported experiences of 8 types of mistreatment. Survey-weighted rates of any mistreatment and each mistreatment indicator were estimated, and survey-weighted logistic regression models estimated odds ratios (ORs) and 95% CIs.

**Results:**

The sample included 4458 postpartum individuals representative of 552 045 people who had live births in 2020 in 7 jurisdictions. The mean (SD) age was 29.9 (5.7) years, 2556 (54.4%) identified as White, and 2836 (58.8%) were commercially insured. More than 1 in 8 individuals (13.4% [95% CI, 11.8%-15.1%]) reported experiencing mistreatment during childbirth. The most common type of mistreatment was being “ignored, refused request for help, or failed to respond in a timely manner” (7.6%; 95% CI, 6.5%-8.9%). Factors associated with experiencing mistreatment included being lesbian, gay, bisexual, transgender, queer identifying (unadjusted OR [UOR], 2.3; 95% CI, 1.4-3.8), Medicaid insured (UOR, 1.4; 95% CI, 1.1-1.8), unmarried (UOR, 0.8; 95% CI, 0.6-1.0), or obese before pregnancy (UOR, 1.3; 95% CI, 1.0-1.7); having an unplanned cesarean birth (UOR, 1.6; 95% CI, 1.2-2.2), a history of substance use disorder (UOR, 2.6; 95% CI, 1.3-5.1), experienced intimate partner or family violence (UOR, 2.3; 95% CI, 1.3-4.2), mood disorder (UOR, 1.5; 95% CI, 1.1-2.2), or giving birth during the COVID-19 public health emergency (UOR, 1.5; 95% CI, 1.1-2.0). Associations of mistreatment with race and ethnicity, age, educational level, rural or urban geography, immigration status, and household income were ambiguous.

**Conclusions and Relevance:**

This cross-sectional study of individuals who had a live birth in 2020 in 6 states and New York City found that mistreatment during childbirth was common. There is a need for patient-centered, multifaceted interventions to address structural health system factors associated with negative childbirth experiences.

## Key Points

**Question:**

How often do birthing individuals in the US experience mistreatment by health care professionals during childbirth?

**Findings:**

In this cross-sectional study, 13.4% of birthing individuals reported experiencing mistreatment during childbirth. Individuals at higher risk included those who were unmarried; were Medicaid insured; were lesbian, gay, bisexual, transgender, queer identifying; had obesity; had a history of substance use disorder, mood disorders, or intimate partner or family violence; or had an unplanned cesarean birth.

**Meaning:**

These results suggest that structural social stigmas permeate the birth experience and shape how care is received, highlighting the need for patient-centered interventions to improve childbirth experiences.

## Teaser

This cross-sectional study conducted in 6 states and New York City estimates the prevalence of mistreatment by health care professionals during childbirth among a representative multistate sample and identifies patient characteristics associated with mistreatment experiences.

## Introduction

Discrimination and lack of respectful care are thought to be key factors associated with disparities in maternal mortality and morbidity in the US.^1,2^ Negative experiences during childbirth can have long-term consequences for birthing individuals, including posttraumatic stress disorder, negative body image, feelings of dehumanization, and changes in future reproductive decisions.^3,4,5,6,7^ However, experiences of mistreatment during childbirth have not been widely documented in the US. In 2019, the Giving Voice to Mothers (GVtM) Study developed the Mistreatment by Care Providers in Childbirth (MCPC) scale, the first patient-designed and validated measure of self-reported mistreatment during childbirth in the US, to our knowledge.^7^ Based on a convenience sample of birthing individuals from marginalized groups, the GVtM study found that 17% experienced mistreatment. Rates were higher among members of racial and ethnic minority populations and individuals with low socioeconomic status.^7^ In 2023, the Centers for Disease Control and Prevention (CDC) measured mistreatment among a convenience sample of mothers with children younger than 18 years; 20% reported mistreatment during pregnancy and delivery, with higher rates among Black (30%), Hispanic (29%), and publicly insured mothers (26%).^8^

Although these studies suggest that mistreatment during pregnancy and delivery is common in the US, both relied on convenience samples. The present study uses a large, representative, multistate sample to (1) estimate the prevalence of mistreatment by health care professionals during childbirth, (2) identify the most common types of mistreatment, and (3) identify the demographic, social, and clinical characteristics associated with mistreatment experiences.

## Methods

### Data and Sample

For this cross-sectional study, we used data from the 2020 Postpartum Assessment of Health Survey (PAHS), a multistate survey of birthing individuals 12 to 14 months after a live birth in 6 states (Kansas, Michigan, New Jersey, Pennsylvania, Utah, and Virginia) and New York City.^9^ The design of PAHS builds on the CDC Pregnancy Risk and Monitoring System (PRAMS). The participating jurisdictions were selected based on PRAMS sample size, meeting CDC PRAMS response rate thresholds, and their willingness and capacity to collaborate in the PAHS.^9^ Each year, the PRAMS sampling frame comprises a stratified random sample of live births drawn monthly from state or city birth certificates.^10^ Individuals in the 2020 PRAMS sampling frame gave birth between January and December 2020 and completed the PRAMS survey from 2 to 6 months post partum. PRAMS respondents were given the option to opt out of being contacted again for the PAHS from 12 to 14 months post partum. Verbal or written consent was obtained depending on the mode of the survey response. PAHS recruitment and data collection then occurred from January 1, 2021, to March 31, 2022. Of those contacted for the PAHS (6021 of 8473; 71.1% of 2020 PRAMS respondents), 4598 completed the survey (76.4% response rate). The PAHS was offered in English and Spanish. Individual-level PAHS responses were linked to PRAMS responses and birth certificate variables. This study was approved by the institutional review boards of the CDC, Columbia University, Rutgers University, and each local jurisdiction. This study followed the Strengthening the Reporting of Observational Studies in Epidemiology (STROBE) reporting guideline.

### Outcome

The primary outcome was any mistreatment. The MCPC scale was developed and validated using a community-based participatory research process with targeted recruitment of pregnant individuals from racial and ethnic minority populations.^7^ PAHS respondents were asked to think back to their birthing experience in the previous year to recall whether they had experienced any of 7 issues or behaviors from health care professionals during childbirth (eTable 1 in Supplement 1), which included physical abuse, verbal abuse (shouted at or scolded, or threatened), neglect, abandonment, lack of informed consent, and breach of confidentiality. Although not included in the MCPC scale, PAHS respondents could also indicate that they experienced any other mistreatment. Any mistreatment was coded as 1 if respondents answered yes to any of the 7 issues or any other mistreatment and 0 if respondents answered no to all.

### Covariates

We measured individual-level demographic, social, and clinical characteristics that have been associated with patients’ perinatal or other health care experiences in prior research. Self-reported race and ethnicity, which was shown to be a factor associated with mistreatment in the GVtM study, was measured on the PAHS. Respondents could choose between 8 categories: Asian; Black; Hispanic or Latinx; Native Hawaiian or Pacific Islander; Native American or Alaska Native; Southwest Asian, Middle Eastern, or North African; White; and multiple minoritized races. Most respondents (95.0% [4368 of 4596]) selected a single race and ethnicity category. We categorized individuals who selected 2 races and ethnicities, including White, as the other race and ethnicity, and those who selected multiple races and ethnicities other than White as “multiple minoritized races.”

Other sociodemographic characteristics included age; lesbian, gay, bisexual, transgender, queer (LGBTQ) identity; marital or domestic partner status; educational level; primary language; household income as a percentage of the 2021 federal poverty level; immigration status (for all jurisdictions except New York City); insurance coverage; and rural or nonrural geography (based on 2013 Rural-Urban Continuum Codes).^11^ Missingness for sociodemographic variables measured in the PAHS was low (range, 0.1%-4.4%). For variables with an equivalent measure in the PRAMS or the birth certificate (race and ethnicity, age, marital status, educational level, income, and insurance at birth), we used these measures to impute the missing PAHS values.

We drew on prior studies of medical stigma and mistreatment to guide selection of other covariates. Intimate partner violence was associated with mistreatment in the GVtM study.^7^ We therefore measured intimate partner or family violence (IPFV) during or 12 months before pregnancy by one’s husband, current or ex-partner, or another family member. Prior studies have shown that some physical and mental health conditions (eg, excess body weight and behavioral health disorders) carry stigmas that negatively shape health care professionals’ care and attitudes towards patients.^12,13,14^ We measured obesity (body mass index ≥30 [calculated as weight in kilograms divided by height in meters squared]) prior to pregnancy and self-reported diagnosis of the following conditions before or during pregnancy: chronic medical conditions (asthma, diabetes, or hypertension), substance use disorder (SUD) or addiction (excluding smoking or tobacco use), and mood disorders (depression, anxiety, or another mood disorder).

Finally, we included birth characteristics that may shape an individual’s support, autonomy, and ability to participate in shared decision-making,^7,15^ including parity; type of birth (vaginal, planned cesarean delivery, or unplanned cesarean delivery); a composite measure of higher-risk pregnancy (multiple births, preterm birth, gestational diabetes, or gestational hypertension); birth during the COVID-19 public health emergency (from March to December 2020); and presence or absence of a support person during childbirth (ie, no one, current partner or spouse, or others such as an ex-partner or ex-spouse or family members).

### Statistical Analysis

We estimated the survey-weighted rates of any mistreatment and each mistreatment indicator. We used survey-weighted logistic regression models to estimate odds ratios (ORs) and 95% CIs for the association between any mistreatment and patient characteristics. We aimed to identify the groups at highest risk of mistreatment; our goal was not to isolate the independent association of any one factor and mistreatment. We therefore did not adjust for covariates. Acknowledging that stigma and structural discrimination can result from multiple overlapping identities,^16^ we further analyzed the intersectional associations of race and ethnicity with 3 demographic characteristics that had statistically significant associations with mistreatment: marital status, LGBTQ identity, and insurance coverage. To do so, we calculated survey-weighted rates of any mistreatment for groups defined by all 96 combinations of these 4 characteristics. We report rates only for the 29 groups with a total sample size of at least 10 individuals.

All estimates were weighted to be representative of live births in 2020 in the 7 jurisdictions. The PAHS weights accounted for the PRAMS stratified survey design, PRAMS nonresponse, and PAHS nonresponse. The PAHS weights were also calibrated to known population totals by maternal age, race and ethnicity, educational level, marital status, sampling strata, and infant birth weight based on 2020 live birth records in each jurisdiction. Missingness was low for the primary MCPC outcome (3.3%); thus, we conducted complete-case analysis. We conducted statistical tests using 2-sided tests and a significance level of *P* < .05. Analyses were performed using Stata, version 17 (StataCorp).

## Results

Among the sample of 4458 postpartum respondents, representative of 552 045 people who had live births in 2020 in 7 jurisdictions, the mean (SD) age was 29.9 (5.7) years, 2556 (54.4%) identified as White, followed by Hispanic or Latinx (790 [18.3%]), Black (620 [15.0%]), or Asian (319 [8.9%]) (Table 1). Overall, 2695 respondents (58.8%) were between 25 and 34 years of age when they gave birth, 4089 (91.1%) identified as non-LGBTQ, 3571 (78.0%) were married or in a domestic partnership, 3270 (68.5%) had higher than a high school education, and 3787 (82.6%) primarily spoke English. At the time of childbirth, 2836 participants (58.8%) were commercially insured, 1479 (37.9%) were insured by Medicaid, and 142 (3.2%) were uninsured.

**Table 1.** Sample Demographic Characteristics and Rates of Any Mistreatment

Characteristic	Overall, No. (%) (N = 4458)^a^	Any mistreatment (n = 551 [13.4%; 95% CI, 11.8%-15.1%])	
		No. (%)^a^	95% CI, %
Age, y			
18-24	739 (19.4)	110 (14.3)	10.9-18.4
25-29	1220 (26.3)	163 (16.3)	12.8-20.7
30-34	1475 (32.5)	161 (11.8)	9.6-14.4
≥35	1024 (21.9)	117 (11.4)	8.9-14.4
Race and ethnicity			
Asian	319 (8.9)	37 (11.5)	8.2-15.9
Black	620 (15.0)	98 (15.9)	12.1-20.6
Hispanic or Latinx	790 (18.3)	96 (10.8)	8.4-13.8
Native American or Alaska Native	47 (0.9)	10 (12.5)	3.3-37.5
Native Hawaiian or Pacific Islander	18 (0.2)	NR	NA
SWANA	34 (1.0)	5 (33.7)	13.1-63.2
White	2556 (54.4)	290 (13.3)	11.0-16.0
Multiple minority races	72 (1.4)	14 (16.9)	6.4-37.9
LGBTQ identity			
Non-LGBTQ	4089 (91.1)	482 (12.9)	11.3-14.7
LGBTQ	234 (4.9)	53 (25.1)	16.8-35.7
Missing	135 (4.0)	16 (10.2)	4.8-20.4
Marital status			
Not married	887 (22.0)	143 (16.0)	13.0-19.6
Married or domestic partnership	3571 (78.0)	408 (12.6)	11.0-14.5
Education			
<High school	312 (9.0)	37 (10.0)	6.3-15.6
High school	876 (22.6)	114 (15.2)	11.4-19.9
>High school	3270 (68.5)	400 (13.2)	11.5-15.1
Primary language			
English	3787 (82.6)	473 (13.9)	12.0-15.9
Spanish	388 (10.0)	34 (8.0)	5.2-12.1
Other	271 (7.4)	43 (15.9)	10.8-22.8
Immigration status			
US citizen or lawful permanent resident	3539 (77.4)	417 (13.0)	11.1-15.2
Neither or other	217 (5.7)	23 (11.9)	6.6-20.6
Missing^b^	702 (16.9)	111 (15.7)	13.0-18.8
Household income (ie, FPL), %			
<100	1086 (26.2)	155 (14.0)	11.0-17.6
100-199	900 (19.9)	127 (15.1)	11.5-19.5
200-399	1288 (27.5)	151 (14.7)	11.7-18.3
≥400%	1044 (22.0)	100 (10.0)	7.7-13.0
Missing	140 (4.4)	18 (10.3)	5.5-18.4
Insurance at birth			
Commercial, military, or other	2836 (58.8)	310 (12.0)	10.1-14.1
Medicaid or other public	1479 (37.9)	230 (16.2)	13.5-19.2
Uninsured	142 (3.2)	11 (6.3)	3.0-12.7
Geography			
Urban	3890 (88.5)	494 (13.7)	12.1-15.4
Rural	517 (10.2)	46 (9.8)	5.4-17.3

Abbreviations: FPL, federal poverty level; LGBTQ, lesbian, gay, bisexual, transgender, queer; NA, not applicable; NR, none reported; SWANA, Southwest Asian, Middle Eastern, or North African.

^a^ Percentages are weighted to be representative of the 7 sample jurisdictions and account for the Postpartum Assessment of Health Survey and Pregnancy Risk and Monitoring System nonresponse and sampling design. Missing category not shown for variables with missing less than 2%.

^b^ Data on immigration status were not collected for respondents from New York City. Excluding New York City, 87 respondents (2.2%) had missing data for immigration status.

### Overall Mistreatment

**Figure 1.**
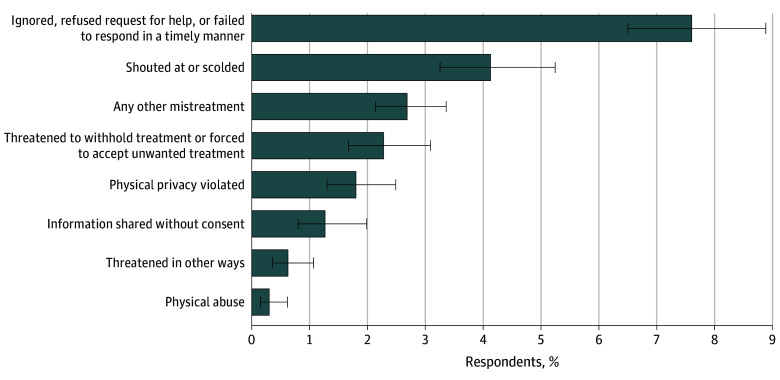
Prevalence of Types of Mistreatment During Childbirth Study measures reflect self-reported responses by postpartum individuals 12 to 14 months after having a live birth. Detailed survey questions are included in eTable 1 in Supplement 1. Percentages and 95% CIs were weighted to be representative of the 7 sample jurisdictions and to account for the Postpartum Assessment of Health Survey and Pregnancy Risk and Monitoring System nonresponse and sampling design. Error bars indicate 95% CIs.

Figure 1 shows rates of mistreatment overall and by type of mistreatment. A total of 13.4% (95% CI, 11.8%-15.1%) of birthing individuals in 2020 reported experiencing some form of mistreatment during childbirth. Being “ignored, refused request for help, or failed to respond in a timely manner” was the most commonly reported type of mistreatment (7.6%; 95% CI, 6.5%-8.9%), followed by being “shouted at or scolded” by health care clinicians (4.1%; 95% CI, 3.3%-5.2%), any other mistreatment (2.7%; 95% CI, 2.1%-3.4%), and having health care clinicians threaten “to withhold treatment or force you to accept treatment that you did not want” (2.3%; 95% CI, 1.7%-3.1%). Mistreatment rates ranged from 9.0% (95% CI, 7.0%-11.5%) in Kansas to 16.9% (95% CI, 13.9%-20.3%) in New York City (eTable 2 in Supplement 1).

### Characteristics Associated With Mistreatment

Rates of mistreatment varied widely by race and ethnicity; however, we did not detect statistically significant differences between White respondents and other groups (Table 2; eTable 3 in Supplement 1). Respondents who were Southwest Asian, Middle Eastern, or North African reported the highest rates of mistreatment (33.7%; 95% CI, 13.1%-63.2%), followed by individuals of multiple minoritized races (16.9%; 95% CI, 6.4%-37.9%), Black respondents (15.9%; 95% CI, 12.1%-20.6%), White respondents (13.3%; 95% CI, 11.0%-16.0%), Native American or Alaska Native respondents (12.5%; 95% CI, 3.3%-37.5%), Asian respondents (11.5%; 95% CI, 8.2%-15.9%), and Hispanic or Latinx respondents (10.8%; 95% CI, 8.4%-13.8%) (Table 1).

**Table 2.** Sample Social and Clinical Characteristics and Rates of Any Mistreatment

Characteristic	Overall, No. (%) (N = 4458)^a^	Any mistreatment (n = 551 [13.4%; 95% CI, 11.8%-15.1%])	
		No. (%)^a^	95% CI
Social and behavioral risks			
Prenatal smoking^b^			
Did not smoke	4172 (94.5)	495 (13.4)	11.7-15.2
Smoked during pregnancy	256 (5.5)	50 (14.1)	8.8-21.9
Substance use disorder^c^			
No substance use	4349 (97.9)	527 (13.1)	11.5-14.8
Substance use	101 (2.1)	24 (28.1)	16.9-43.0
Intimate partner or family violence^c^			
No intimate partner or family violence	4280 (97.2)	510 (13.1)	11.4-14.9
Intimate partner or family violence	137 (2.8)	37 (26.1)	16.8-38.1
Pregnancy characteristics			
Prepregnancy chronic physical conditions^d^			
None	3711 (83.3)	433 (13.2)	11.4-15.2
Asthma, diabetes, or hypertension	747 (16.7)	118 (14.3)	11.0-18.3
Mood disorders^c^			
None	3320 (77.1)	366 (12.2)	10.4-14.2
Depression or anxiety	1120 (22.9)	184 (17.6)	13.8-22.1
Nulliparous			
≥1 Previous live births	2655 (59.7)	301 (12.5)	10.4-14.9
0 Previous live births	1800 (40.3)	249 (14.8)	12.6-17.2
Obesity^e^			
Not obese	3173 (71.7)	382 (12.5)	10.7-14.5
Obese	1224 (28.3)	161 (15.6)	13.0-18.6
Birth characteristics			
Higher-risk pregnancy^f^			
None	2879 (75.6)	364 (13.6)	11.7-15.8
Higher-risk pregnancy	1579 (24.4)	187 (12.6)	10.1-15.5
Delivery type			
Vaginal	2917 (69.6)	357 (12.9)	11.0-15.2
Planned cesarean birth	696 (16.0)	63 (10.1)	6.9-14.5
Unplanned cesarean birth	833 (14.4)	130 (19.3)	15.5-23.9
Birth during COVID-19 public health emergency			
Born before March 2020	1198 (28.6)	121 (10.5)	8.1-13.5
Born after March 2020	3260 (71.4)	430 (14.5)	12.7-16.5
Support at birth			
Current partner or spouse	3734 (83.1)	423 (12.6)	10.9-14.4
Other (ex-partner or ex-spouse, family, or other)	465 (12.2)	76 (16.6)	11.7-23.0
No one	240 (4.7)	45 (18.9)	11.9-28.9

^a^ Percentages are weighted to be representative of the 7 sample jurisdictions and account for the Postpartum Assessment of Health Survey and Pregnancy Risk and Monitoring System nonresponse and sampling design. Missing category not shown for variables with missing less than 2%.

^b^ Prenatal smoking assessed during pregnancy.

^c^ Before or during pregnancy.

^d^ Prepregnancy chronic physical conditions included asthma, diabetes, or hypertension.

^e^ Obesity was defined as body mass index of 30 or greater (calculated as weight in kilograms divided by height in meters squared) prior to pregnancy.

^f^ Higher-risk pregnancy included multiple births, preterm birth, gestational diabetes, or gestational hypertension.

**Figure 2.**
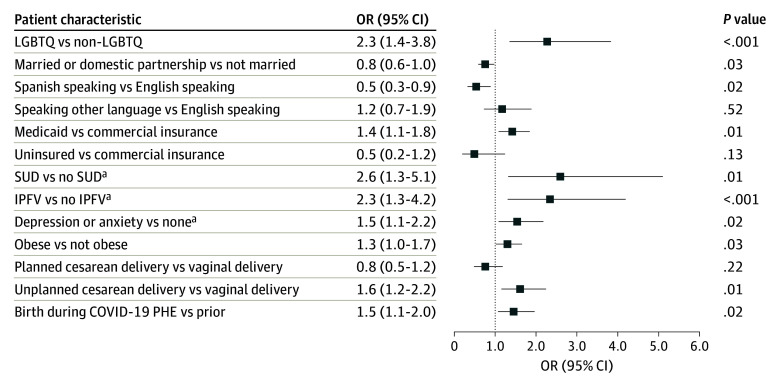
Unadjusted Associations Between Any Mistreatment and Patient Characteristics Study measures reflect self-reported responses by postpartum individuals 12 to 14 months after having a live birth. Odds ratios (ORs) and 95% CIs were weighted to be representative of the 7 sample jurisdictions and to account for the Postpartum Assessment of Health Survey and Pregnancy Risk and Monitoring System nonresponse and sampling design. *P* values indicate the statistical significance of differences in the odds of reporting any item on the Mistreatment by Care Providers During Childbirth scale relative to the reference group based on unadjusted survey-weighted logistic regressions. Only characteristics with statistically significant comparisons are shown. IPFV indicates intimate partner or family violence; LGBTQ, lesbian, gay, bisexual, transgender, queer; PHE, public health emergency; and SUD, substance use disorder. ^a^Before or during pregnancy.

Figure 2 shows the unadjusted ORs (UORs) for patient characteristics that were statistically significantly associated with any mistreatment. We found that LGBTQ respondents were twice as likely to experience any mistreatment compared with non-LGBTQ respondents (UOR, 2.3; 95% CI, 1.4-3.8). Odds of mistreatment were higher among those nsured by Medicaid at birth (UOR, 1.4; 95% CI, 1.1-1.8) and lower among respondents who were married relative to those who were not married or in a domestic partnership (UOR, 0.8; 95% CI, 0.6-1.0). Spanish language speakers were less likely to report mistreatment relative to primary English speakers (UOR, 0.5; 95% CI, 0.3-0.9). We did not identify statistically significant differences in mistreatment rates by age, educational level, rural or urban geography, immigration status, or household income (eTable 3 in Supplement 1).

Individuals with a history of SUD (UOR, 2.6; 95% CI, 1.3-5.1) and IPFV (UOR, 2.3; 95% CI, 1.3-4.2) were nearly twice as likely to report mistreatment relative to those without SUD or IPFV (Figure 2). Respondents with mood disorders before or during pregnancy (UOR, 1.5; 95% CI, 1.1-2.2) and those who were obese prior to pregnancy (UOR, 1.3; 95% CI, 1.0-1.7) were also more likely to report mistreatment. Respondents with an unplanned cesarean birth (UOR, 1.6; 95% CI, 1.2-2.2) reported higher rates of mistreatment relative to those with a vaginal birth, as did those who gave birth during the COVID-19 public health emergency (UOR, 1.5; 95% CI, 1.1-2.0). We did not find statistically significant associations of mistreatment with support at childbirth, chronic physical conditions, parity, or higher-risk pregnancy (eTable 3 in Supplement 1).

The most common forms of mistreatment differed by patient characteristics (eTable 5 in Supplement 1). For example, LGBTQ individuals reported statistically significantly higher rates of being “threatened [with] withhold[ing] treatment or forced to accept [unwanted] treatment” compared with non-LGBTQ individuals (11.1% vs 1.9%). Southwest Asian, Middle Eastern, or North African respondents were more likely to report that their “physical privacy was violated, such as being uncovered or having people in the delivery room” without consent relative to White respondents (21.3% vs. 1.2%).

### Intersectionality Between Mistreatment and Demographic Characteristics

eTable 4 in Supplement 1 shows the rates of mistreatment by combinations of race and ethnicity, marital status, LGBTQ identity, and insurance at time of birth among sample sizes larger than 10. Across Black and White groups, the combination of identifying as LGBTQ, being unmarried, and Medicaid insured was associated with higher risk of mistreatment, with more than one-third of respondents with these intersecting identities reporting mistreatment (Black, 36.1%; 95% CI, 11.7%-70.5%; White, 36.2%; 95% CI, 9.7%-75.0%). Southwest Asian, Middle Eastern, or North African respondents who were Medicaid insured, married or in a domestic partnership, and non-LGBTQ identifying reported the highest rate of mistreatment (55.9%; 95% CI, 13.1%-91.4%); however, the 95% CIs were wide.

## Discussion

Using multistate representative survey data, we found that mistreatment by health care professionals during childbirth is a common experience in the US, affecting more than 1 in 8 individuals with a live birth in 2020. The highest rates of mistreatment occurred among individuals who were unmarried; Medicaid insured; LGBTQ identifying; obese; had a history of SUD, mood disorders, or IPFV; and those who had an unplanned cesarean birth.

The overall prevalence of mistreatment in our sample is lower than in the GVtM study (17.4% in a convenience sample drawn from marginalized communities^7^) and the CDC survey (20% in an online convenience sample of mothers with children <18 years).^8^ However, the prevalence rate in our study of 13.4%—representative of all birthing individuals in 6 states and New York City—suggests a need for interventions to improve respectful maternity care in the US. Similar to the CDC study, which did not conduct statistical testing, we found higher rates of mistreatment among Black and multiracial individuals, as well as those with public insurance. Similarities with the GVtM study include statistically significantly higher mistreatment rates among individuals with a history of SUD or IPFV, those with public insurance, and those with unplanned cesarean births. We also isolated risk factors not previously explored, including LGBTQ identity, obesity, mood disorders, and marital status.

Many of our results suggest that a pervasive structural social stigma permeates the birth experience and shapes how care is received.^17^ For example, we found that LGBTQ-identifying individuals were twice as likely to experience mistreatment, associated with higher rates of feeling forced to accept unwanted treatment or being denied wanted treatment. These findings align with prior work demonstrating poorer birth outcomes among sexual minority women,^18^ as well as research linking stigma and heterosexist policies to minority stress and adverse health outcomes among LGBTQ-identifying individuals.^19^

Similarly, our results follow prior research that has linked stigma, discrimination in health care settings, and adverse health outcomes for people with excess body weight,^20,21^ birthing individuals who are unmarried,^22,23,24,25^ and individuals of low socioeconomic status who are publicly insured.^26^ The high prevalence of mistreatment experienced by Medicaid-insured birthing individuals warrants attention, and Medicaid program administrators could explore options such as coverage for doulas and financial incentives to encourage respectful maternity care.

Our findings of increased mistreatment among patients with SUD, mood disorders, and a history of IPFV are concerning. Recent research has found that homicide, suicide, and drug overdose are leading causes of deaths after childbirth.^27,28^ Mistreatment during childbirth may deter patients from seeking potentially lifesaving health care services, such as care for mental health, substance use, and experiences of IPFV. Mistreatment could also affect patients’ trust in health care professionals and affiliated institutions, with adverse long-term consequences for care seeking, disclosure to clinicians, and uptake of social services. Health care professionals and institutions could adopt targeted interventions to address the needs of at-risk patient groups, to foster inclusive and justice-informed care, and to actively discourage, make visible, and remedy discrimination against patients.^29,30^

Unlike the GVtM study, we did not identify statistically significant associations of mistreatment with younger age (17-25 years), race and ethnicity, nulliparity, or having a high-risk pregnancy. The GVtM study found that Black, Hispanic, and Indigenous mothers were statistically significantly more likely than White mothers to experience mistreatment. In our study, Southwest Asian, Middle Eastern, or North African respondents were the most likely to report mistreatment, followed by Black individuals and people of multiple minoritized races. Group differences, however, were not statistically significant, which may be due to sample size and use of survey weighting, which reduces statistical power but allows for representative estimates.

Numerous studies have identified control—namely, participation in shared decision-making and patient-clinician communication, including managing complications—as one of the most important factors in birth satisfaction.^4,31,32^ The high rate of mistreatment experienced by respondents with unplanned cesarean births may reflect the dynamics of patient disempowerment, such as loss of autonomy and lack of communication regarding the indication for the procedure. Reported mistreatment among those with unplanned cesarean births was largely driven by high rates of being forced to accept unwanted treatment or being threatened with withholding treatment.

This study also points to some conditions of care settings that might be associated with mistreatment experiences. Mistreatment was statistically significantly higher among respondents who gave birth in the months after the onset of the COVID-19 pandemic, when health care systems and personnel were experiencing extreme stress and resource scarcity. Health care professionals, staff members, facilities, and patients were all navigating a lack of vaccines, a shortage in personal protective equipment, and significant changes to hospital visitor policies that reduced and, in some cases, briefly eliminated support persons during labor and childbirth. Other studies have shown how the health, social, and policy contexts of the pandemic were adversely associated with maternity care and patient experiences.^33,34,35^ The high proportions of respondents in our study who reported being “ignored,” being “refused requests for help,” and being “shouted at or scolded” may partly reflect clinician burnout and resource constraints.

### Limitations

This study has some limitations. First, while our findings were representative of the 7 included jurisdictions, which comprised 19.8% of US births in 2020,^36^ the results may not be generalizable to all US jurisdictions. Second, some null results, including comparisons by race and ethnicity and immigration status (which was not collected in New York City), could be due to insufficient statistical power rather than lack of true differences. Third, “any other mistreatment” was the third most common form of mistreatment, suggesting that some perceived mistreatment experiences are not captured by the options in the MCPC scale. Incorporating the MCPC scale in larger population surveys, such as the PRAMS, with a free text option, would allow for the tracking of mistreatment rates across jurisdictions and over time, the exploration of other forms of mistreatment, and the statistical power to better evaluate disparities. Fourth, the MCPC scale does not capture mistreatment during pregnancy. Fifth, all variables are self-reported and could be subject to recall or social desirability bias (eg, reporting of SUD). Sixth, PAHS participants gave birth in 2020. Mistreatment rates could vary outside of the pandemic context. Seventh, while the PAHS was offered in Spanish, we found considerably lower rates of mistreatment among Spanish-speaking individuals. This finding could reflect a real difference or differences in the interpretation of the MCPC scale, which was not specifically validated for Spanish populations.

## Conclusions

In this cross-sectional study conducted in 6 states and New York City, we found that mistreatment during childbirth was a common experience. To our knowledge, evidence of effective interventions to improve respectful maternity care in the US is scant. There is a need for the development and evaluation of patient-centered, multifaceted interventions that address implicit biases, cultural competence, health care workforce conditions, the inclusivity of clinical settings, and other structural factors, including health system factors, to improve childbirth experiences.

## Notes

Supplement 1. **eTable 1.** Mistreatment by Care Providers in Childbirth (MCPC) Survey Instrument **eTable 2.** Sample Characteristics by Jurisdiction and Rates of Any Mistreatment **eTable 3.** Unadjusted Associations Between Any Mistreatment and Patient Characteristics **eTable 4.** Percentage of Respondents Experiencing Any Mistreatment by Race-Ethnicity, Marital Status, Sexual Orientation, and Insurance Status, for Combinations With Sample Size n ≥ 10 **eTable 5.** Individual Types of Mistreatment by Patient Characteristics

Supplement 2.
Data Sharing Statement
