# Supplementary material for: Disparities in Mistreatment During Childbirth
Source: JAMA Netw Open. 2024 Apr 4;7(4):e244873. doi: 10.1001/jamanetworkopen.2024.4873 (PMC11192180; doi:10.1001/jamanetworkopen.2024.4873)
Supplement: Supplement 1. — eTable 1. Mistreatment by Care Providers in Childbirth (MCPC) Survey Instrument eTable 2. Sample Characteristics by Jurisdiction and Rates of Any Mistreatment eTable 3. Unadjusted Associations Between Any Mistreatment and Patient Characteristics eTable 4. Percentage of Respondents Experiencing Any Mistreatment by Race-Ethnicity, Marital Status, Sexual Orientation, and Insurance Status, for Combinations With Sample Size n ≥ 10 eTable 5. Individual Types of Mistreatment by Patient Characteristics [file jamanetwopen-e244873-s001.pdf]

Supplementary Online Content

Liu C, Underhill K, Aubey JJ, Samari G, Allen HL, Daw JR. Disparities in mistreatment during childbirth *JAMA Netw Open.* 2024;7(4):e244873. doi:10.1001/jamanetworkopen.2024.4873

eTable 1. Mistreatment by Care Providers in Childbirth (MCPC) Survey Instrument
eTable 2. Sample Characteristics by Jurisdiction and Rates of Any Mistreatment
eTable 3. Unadjusted Associations Between Any Mistreatment and Patient Characteristics
eTable 4. Percentage of Respondents Experiencing Any Mistreatment by Race-Ethnicity, Marital Status, Sexual Orientation, and Insurance Status, for Combinations With Sample Size n ≥ 10
eTable 5. Individual Types of Mistreatment by Patient Characteristics

This supplementary material has been provided by the authors to give readers additional information about their work.

eTable 1. Mistreatment by Care Providers in Childbirth (MCPC) Survey Instrument

During your labor and delivery, did you experience any of the following issues or behaviors?(Check all that apply) ...
You experienced physical abuse (including aggressive physical contact, inappropriate sexual conduct, refusal to provide anesthesia for an episiotomy, etc)
Health care clinicians (doctors, midwives, or nurses) shouted at or scolded you
Health care clinicians threatened to withhold treatment or forced you to accept treatment you did not want
Health care clinicians threatened you in any other way
Your private or personal information was shared without your consent
Your physical privacy was violated (e.g., being uncovered or having people in the delivery room without your consent)
Health care clinicians ignored you, refused your request for help, or failed to respond to requests for help in a reasonable amount of time
Any other mistreatment

© 2024 Liu C et al. *JAMA Network Open.*

eTable 2. Sample Characteristics by Jurisdiction and Rates of Any Mistreatment

Jurisdiction	Overall N=4458		Any Mistreatment N=551 (13.4%)		
	N	%	N	%	95% CI
New York City	615	15.0%	107	16.9%	(13.9 - 20.3)
New Jersey	653	15.9%	99	14.8%	(11.6 - 18.7)
Pennsylvania	687	21.5%	84	13.0%	(10.3 - 16.3)
Virginia	624	16.0%	69	11.6%	(7.5 - 17.5)
Michigan	225	17.9%	29	13.5%	(8.9 - 20.0)
Utah	891	7.8%	95	11.2%	(9.0 - 13.9)
Kansas	763	5.8%	68	9.0%	(7.0 - 11.5)

Notes: Statistics shown are unweighted sample sizes, survey-weighted percentages, and 95% confidence intervals. Results are weighted to be representative of the seven sample jurisdictions and account for the PAHS and PRAMS nonresponse and sampling design.

© 2024 Liu C et al. *JAMA Network Open.*

eTable 3. Unadjusted Associations Between Any Mistreatment and Patient Characteristics

Characteristics		Any Mistreatment			P-value
		OR	95% CI Lower	95% CI Upper	
**Demographic Characteristics**					
**Age**	18-24‡	1.0			
	25-29	1.2	0.8	1.8	0.46
	30-34	0.8	0.6	1.1	0.22
	35+	0.8	0.5	1.2	0.24
**Race-Ethnicity**	White‡	1.0			
	Black	1.2	0.8	1.8	0.29
	Hispanic/Latinx	0.8	0.6	1.1	0.17
	Asian	0.8	0.6	1.3	0.44
	SWANA	3.3	0.2	56.4	0.41
	Native American/Alaskan Native	0.9	0.2	4.2	0.92
	Multiple Minority Races	1.3	0.3	5.9	0.71
**LGBTQ Identity**	Non-LGBTQ‡	1.0			
	LGBTQ	2.3	1.4	3.8	0.00**
**Marital Status**	Not Married‡	1.0			
	Married/Domestic partner	0.8	0.6	1.0	0.03**
**Education**	<High School‡	1.0			
	High School	1.6	0.8	3.1	0.15
	>High School	1.4	0.8	2.5	0.30
**Primary Language**	English‡	1.0			
	Spanish	0.5	0.3	0.9	0.02**
	Other	1.2	0.7	1.9	0.52
**Immigration Status***	US Citizen/PR/Green Card‡	1.0			
	Neither/other	0.9	0.4	1.9	0.78
**Household Income**	<100% FPL‡	1.0			
	100-199%	1.1	0.7	1.6	0.65
	200-399%	1.1	0.7	1.6	0.76
	400%+	0.7	0.5	1.0	0.05
**Insurance at birth**	Commercial/Military/Other‡	1.0			
	Medicaid or other public	1.4	1.1	1.8	0.01**
	Uninsured	0.5	0.2	1.2	0.13
**Geography**	Non-Rural‡	1.0			
	Rural	0.7	0.3	1.4	0.29
**Social Characteristics**					
**Prenatal Smoking**	Didn't Smoke‡	1.0			
	Smoked during Pregnancy	1.1	0.6	1.9	0.82
**Substance Use Disorder†**	No Substance Use‡	1.0			
	Substance Use	2.6	1.3	5.1	0.01**
**Intimate Partner/Family Violence†**	No IPFV‡	1.0			
	IPFV	2.3	1.3	4.2	0.00**
**Clinical Characteristics**					
**Pre-pregnancy Chronic Physical Conditions**	None‡	1.0			
	Asthma/Diabetes/Hypertension	1.1	0.8	1.6	0.59
**Mood Disorder†**	None‡	1.0			
	Depression/Anxiety	1.5	1.1	2.2	0.02**
**Nulliparous**	>=1 Prev. Live Birth‡	1.0			
	0 Prev. Live Birth	1.2	0.9	1.6	0.16
**Obesity**	Not Obese‡	1.0			
	Obese	1.3	1.0	1.7	0.03**

© 2024 Liu C et al. *JAMA Network Open.*

**Birth Characteristics**					
**Higher Risk Pregnancy**	Not High Risk Pregnancy[‡]	1.0			
	Higher Risk Pregnancy	0.9	0.7	1.2	0.57
**Delivery Type**	Vaginal[‡]	1.0			
	Planned Cesarean	0.8	0.5	1.2	0.22
	Unplanned Cesarean	1.6	1.2	2.2	0.01**
**Birth during COVID-19 PHE**	Jan-Feb 2020[‡]	1.0			
	March-Dec 2020	1.5	1.1	2.0	0.02**
**Support at Birth**	Current Partner/Spouse[‡]	1.0			
	Other (Ex/Family/Other)	1.4	0.9	2.1	0.15
	No One	1.6	0.9	2.9	0.11

Notes: Results shown are unadjusted survey-weighted odds ratios and 95% confidence intervals. P-values represent the statistical significance of differences in odds of experiencing mistreatment for each patient characteristic relative to the [‡]reference group. *Immigration status was not collected for respondents from NYC. SWANA = Southwest Asian, Middle Eastern or North African. NHPI not presented as no respondents reported mistreatment. Prenatal smoking assesses smoking during pregnancy. Pre-pregnancy Chronic Physical Conditions include asthma, diabetes, or hypertension. Obesity defined as BMI>=30. Higher risk pregnancy includes multiple births, preterm birth, gestational diabetes, or gestational hypertension. ‡Reference group. †Before/during pregnancy. **p<0.05.

© 2024 Liu C et al. *JAMA Network Open.*

eTable 4. Percentage of Respondents Experiencing Any Mistreatment by Race-Ethnicity, Marital Status, Sexual Orientation, and Insurance Status, for Combinations With Sample Size n ≥ 10

LGBTQ Identity	Marital Status	Insurance at Birth	Race-Ethnicity	Total Sample Size	# Reporting Mistreatment	Any Mistreatment (Weighted %)	95% CI
non-LGBTQ	Married/DP	Commercial/Military/Other	White	1871	178	10.8%	(8.7 - 13.4)
non-LGBTQ	Married/DP	Medicaid/Other Public	White	332	39	15.8%	(10.2 - 23.8)
non-LGBTQ	Married/DP	Commercial/Military/Other	H/L	242	27	11.5%	(7.0 - 18.1)
non-LGBTQ	Married/DP	Medicaid/Other Public	H/L	236	20	8.6%	(5.0 - 14.3)
non-LGBTQ	Married/DP	Commercial/Military/Other	Asian	235	26	11.9%	(8.1 - 17.2)
non-LGBTQ	Not Married	Medicaid/Other Public	Black	231	36	16.4%	(10.2 - 25.2)
non-LGBTQ	Not Married	Medicaid/Other Public	White	154	33	23.2%	(14.2 - 35.5)
non-LGBTQ	Married/DP	Commercial/Military/Other	Black	142	18	16.4%	(7.9 - 31.2)
non-LGBTQ	Not Married	Medicaid/Other Public	H/L	141	21	15.7%	(9.6 - 24.6)
non-LGBTQ	Married/DP	Medicaid/Other Public	Black	123	24	12.3%	(6.3 - 22.7)
non-LGBTQ	Not Married	Commercial/Military/Other	Black	77	7	9.4%	(3.8 - 21.3)
LGBTQ	Married/DP	Commercial/Military/Other	White	76	12	20.0%	(8.2 - 41.4)
non-LGBTQ	Not Married	Commercial/Military/Other	White	74	9	9.9%	(3.4 - 25.4)
non-LGBTQ	Married/DP	Uninsured	H/L	47	5	4.7%	(1.2 - 16.3)
non-LGBTQ	Married/DP	Medicaid/Other Public	Asian	42	6	14.1%	(4.9 - 34.1)
non-LGBTQ	Not Married	Commercial/Military/Other	H/L	39	8	16.9%	(6.6 - 37.0)
non-LGBTQ	Married/DP	Uninsured	White	38	3	11.1%	(2.5 - 37.6)
non-LGBTQ	Married/DP	Commercial/Military/Other	MMR	26	4	20.1%	(3.9 - 60.8)
LGBTQ	Married/DP	Medicaid/Other Public	White	24	4	30.5%	(9.0 - 66.0)
LGBTQ	Not Married	Medicaid/Other Public	Black	21	5	36.1%	(11.7 - 70.5)
LGBTQ	Not Married	Medicaid/Other Public	White	21	5	36.2%	(9.7 - 75.0)
non-LGBTQ	Married/DP	Commercial/Military/Other	SWANA	18	1	20.4%	(2.7 - 70.2)
non-LGBTQ	Not Married	Uninsured	H/L	17	1	14.0%	(1.2 - 68.7)
non-LGBTQ	Not Married	Medicaid/Other Public	MMR	16	3	11.5%	(2.1 - 43.8)
non-LGBTQ	Married/DP	Commercial/Military/Other	NA/AN	13	2	14.8%	(1.2 - 71.6)
non-LGBTQ	Married/DP	Medicaid/Other Public	NA/AN	13	2	11.6%	(0.1 - 94.3)
non-LGBTQ	Married/DP	Medicaid/Other Public	MMR	11	2	4.4%	(0.2 - 46.9)
non-LGBTQ	Married/DP	Medicaid/Other Public	SWANA	10	3	55.9%	(13.1 - 91.4)
non-LGBTQ	Married/DP	Commercial/Military/Other	NHPI	10	NR	0.0%	

© 2024 Liu C et al. *JAMA Network Open.*

Caption: Study measures reflect self-reported responses by postpartum people 12-14 months after having a live birth. Statistics shown are unweighted sample sizes, percentages that are weighted to be representative of the seven sample jurisdictions and account for the PAHS and PRAMS nonresponse and sampling design, and 95% confidence intervals. Combinations that comprise fewer than 10 respondents are suppressed due to large standard errors and unreliable estimates. LGBTQ= Lesbian, Gay, Bisexual, Transgender, Queer. Married/DP = Married or living with a domestic partner. SWANA= Southwest Asian, Middle Eastern or North African; NHPI=Native Hawaiian or Pacific Islander; NA/AN=Native American or Alaskan Native. MMR = Multiple Minority Races. NR = None Reported.

© 2024 Liu C et al. *JAMA Network Open.*

eTable 5. Individual Types of Mistreatment by Patient Characteristics

Subgroups	MCPC Ignore		MCPC Shout		MCPC Other		MCPC Force		MCPC Privacy		MCPC Info		MCPC Threat		MCPC Abuse		
	**%**	**(95% CI)**	**%**	**(95% CI)**	**%**	**(95% CI)**	**%**	**(95% CI)**	**%**	**(95% CI)**	**%**	**(95% CI)**	**%**	**(95% CI)**	**%**	**(95% CI)**	**N**
Total Sample	7.6	(6.5 - 8.9)	4.1	(3.3 - 5.2)	2.7	(2.1 - 3.4)	2.3	(1.7 - 3.1)	1.8	(1.3 - 2.5)	1.3	(0.8 - 2.0)	0.6	(0.4 - 1.1)	0.3	0.3	4458
**Race-Ethnicity**																	
White	8.1	(6.4 - 10.3)	3.7	(2.6 - 5.1)	2.5	(1.7 - 3.7)	2.2	(1.4 - 3.4)	1.2	(0.8 - 1.7)	1.2	(0.6 - 2.2)	0.6	(0.2 - 1.4)	0.3	(0.1 - 0.6)	2556
Black	7.6	(5.2 - 10.9)	7.8	(5.0 - 12.0)	3.3	(1.9 - 5.7)	3.4	(2.1 - 5.6)	2.9	(1.6 - 5.3)	2.0	(1.0 - 4.1)	1.2	(0.4 - 3.3)	0.1	(0.0 - 0.3)	620
Hispanic/Latinx	6.6	(4.7 - 9.0)	2.4	(1.4 - 4.1)	2.2	(1.3 - 3.9)	1.3	(0.7 - 2.4)	2.0	(1.0 - 3.7)	1.0	(0.4 - 2.2)	0.5	(0.1 - 1.6)	0.4	(0.1 - 1.9)	790
Asian	5.9	(3.8 - 9.2)	3.6	(1.8 - 7.3)	3.3	(1.5 - 7.2)	1.1	(0.4 - 3.5)	1.2	(0.4 - 3.8)	0.2	(0.0 - 1.4)	NR	NR	0.8	(0.2 - 4.0)	319
SWANA	12.7	(3.7 - 35.7)	4.7	(0.5 - 31.1)	4.7	(0.5 - 31.1)	NR	NR	21.3	(5.9 - 53.8)	8.9	(1.0 - 47.8)	NR	NR	NR	NR	34
NAAN	5.3	(0.8 - 29.0)	0.7	(0.1 - 3.4)	1.8	(0.3 - 9.1)	9.9	(2.0 - 37.0)	0.2	(0.0 - 1.8)	0.3	(0.0 - 4.1)	0.3	(0.0 - 4.1)	NR	NR	47
Multiple Minoritized Races	7.2	(2.2 - 21.1)	7.4	(2.2 - 22.4)	5.6	(1.3 - 20.6)	8.6	(1.5 - 36.3)	0.2	(0.1 - 1.2)	NR	NR	4.3	(0.7 - 22.9)	NR	NR	72
**LGBTQ Identity**																	
Non-LGBTQ	7.3	(6.1 - 8.6)	3.9	(3.0 - 5.1)	2.5	(2.0 - 3.3)	1.9	(1.3 - 2.6)	1.7	(1.2 - 2.3)	1.3	(0.8 - 2.0)	0.5	(0.3 - 0.9)	0.3	(0.1 - 0.6)	4089
LGBTQ	14.8	(8.6 - 24.2)	9.4	(5.4 - 16.0)	5.5	(2.6 - 11.2)	11.1	(5.7 - 20.5)	3.0	(1.4 - 6.3)	2.3	(0.8 - 6.5)	2.4	(0.5 - 11.7)	1.2	(0.3 - 5.1)	234
**Marital Status**																	
Not Married	9.5	(7.0 - 12.6)	5.9	(4.0 - 8.5)	3.4	(2.1 - 5.3)	3.5	(2.1 - 5.7)	2.2	(1.2 - 3.9)	2.1	(1.1 - 3.9)	1.3	(0.5 - 3.0)	0.3	(0.1 - 1.1)	887
Married/Domestic Partner	7.1	(5.9 - 8.5)	3.6	(2.7 - 4.8)	2.5	(1.9 - 3.2)	1.9	(1.3 - 2.8)	1.7	(1.2 - 2.5)	1.1	(0.6 - 1.8)	0.4	(0.2 - 0.9)	0.3	(0.1 - 0.7)	3571
**Primary Language**																	
English	8.3	(6.9 - 9.8)	4.5	(3.5 - 5.8)	2.8	(2.2 - 3.6)	2.4	(1.7 - 3.4)	1.7	(1.2 - 2.4)	1.3	(0.8 - 2.1)	0.6	(0.4 - 1.1)	0.2	(0.1 - 0.5)	3787
Spanish	4.8	(2.8 - 8.0)	1.4	(0.6 - 3.0)	1.9	(0.8 - 4.4)	0.8	(0.3 - 2.3)	0.5	(0.1 - 2.2)	0.5	(0.1 - 1.6)	0.3	(0.0 - 2.0)	0.5	(0.1 - 3.1)	388
Other	4.6	(2.5 - 8.4)	3.6	(1.5 - 8.4)	2.2	(1.1 - 4.3)	2.9	(1.5 - 5.8)	4.9	(2.1 - 10.7)	2.0	(0.5 - 7.3)	1.1	(0.2 - 4.5)	0.7	(0.1 - 5.6)	271
**Insurance at birth**																	
Commercial/Military/Other	6.6	(5.4 - 8.1)	3.8	(2.8 - 5.1)	2.3	(1.7 - 3.1)	1.7	(1.1 - 2.7)	1.2	(0.8 - 1.8)	1.0	(0.5 - 1.8)	0.5	(0.2 - 1.0)	0.3	(0.1 - 0.7)	2836
Medicaid or Other Public	9.6	(7.7 - 12.0)	4.9	(3.4 - 7.1)	3.4	(2.3 - 5.0)	3.2	(2.2 - 4.6)	2.9	(1.8 - 4.5)	1.8	(1.0 - 3.4)	0.9	(0.4 - 2.0)	0.3	(0.1 - 0.9)	1479
Uninsured	2.3	(0.6 - 8.2)	1.8	(0.3 - 11.9)	0.6	(0.1 - 3.4)	1.7	(0.2 - 12.3)	0.8	(0.1 - 6.0)	0.8	(0.1 - 4.6)	NR	NR	NR	NR	142
**Substance use disorder†**																	
No SUD	7.4	(6.3 - 8.7)	3.9	(3.1 - 5.0)	2.7	(2.1 - 3.4)	2.1	(1.6 - 2.9)	1.8	(1.3 - 2.5)	1.3	(0.8 - 2.0)	0.5	(0.3 - 0.8)	0.3	(0.2 - 0.6)	4349
SUD	17.5	(8.9 - 31.5)	13.4	(6.0 - 27.1)	3.1	(1.1 - 8.4)	9.0	(2.7 - 25.9)	1.5	(0.5 - 4.1)	1.5	(0.3 - 6.1)	6.7	(1.4 - 25.8)	0.3	(0.0 - 2.3)	101
**Intimate Partner/Family Violence†**																	
No IPFV	7.3	(6.2 - 8.6)	3.9	(3.0 - 5.0)	2.6	(2.1 - 3.3)	2.2	(1.6 - 3.0)	1.8	(1.3 - 2.5)	1.2	(0.8 - 2.0)	0.6	(0.3 - 1.1)	0.3	(0.1 - 0.6)	4280
IPFV	18.8	(10.3 - 31.9)	13.5	(6.5 - 25.9)	6.1	(2.4 - 14.6)	6.2	(2.3 - 15.4)	2.3	(0.8 - 6.6)	2.8	(0.6 - 12.2)	0.9	(0.1 - 6.8)	1.9	(0.3 - 11.3)	137
**Mood Disorder†**																	
None	6.9	(5.6 - 8.3)	3.3	(2.5 - 4.4)	2.2	(1.6 - 3.0)	2.0	(1.4 - 2.8)	1.8	(1.2 - 2.7)	1.1	(0.7 - 1.9)	0.6	(0.3 - 1.1)	0.2	(0.1 - 0.5)	3320
Depression/Anxiety	10.1	(7.7 - 13.2)	6.8	(4.2 - 10.8)	4.4	(2.8 - 6.8)	3.3	(2.0 - 5.4)	1.7	(1.1 - 2.9)	1.7	(0.7 - 4.2)	0.7	(0.3 - 1.9)	0.7	(0.3 - 1.6)	1120
**Obesity**																	
Not Obese	7.2	(5.8 - 8.9)	3.9	(3.0 - 5.1)	2.5	(2.0 - 3.3)	2.0	(1.4 - 2.9)	1.5	(1.0 - 2.2)	0.7	(0.4 - 1.4)	0.7	(0.4 - 1.3)	0.2	(0.1 - 0.6)	3173
Obese	8.6	(6.7 - 10.9)	4.8	(3.2 - 7.1)	3.2	(2.1 - 4.8)	3.1	(2.0 - 5.0)	2.6	(1.5 - 4.3)	2.7	(1.4 - 5.2)	0.5	(0.2 - 1.4)	0.3	(0.2 - 0.8)	1224
**Delivery Type**																	

© 2024 Liu C et al. *JAMA Network Open.*

Vaginal	7.1	(5.9 - 8.6)	3.9	(2.9 - 5.3)	2.8	(2.1 - 3.7)	1.8	(1.2 - 2.7)	1.9	(1.3 - 2.7)	1.2	(0.6 - 2.1)	0.5	(0.3 - 0.8)	0.2	(0.1 - 0.6)	2917
Planned Cesarean	6.6	(4.1 - 10.5)	3.4	(1.9 - 6.1)	1.5	(0.7 - 3.0)	1.5	(0.6 - 3.6)	0.9	(0.3 - 2.3)	0.9	(0.3 - 2.6)	1.4	(0.4 - 4.2)	0.5	(0.1 - 2.4)	696
Unplanned Cesarean	11.3	(8.2 - 15.4)	5.9	(3.8 - 9.1)	3.7	(2.2 - 6.2)	5.7	(3.6 - 8.9)	2.5	(1.0 - 5.9)	2.2	(1.1 - 4.4)	0.6	(0.2 - 2.1)	0.5	(0.1 - 1.7)	833
**Birth during COVID-19 PHE**																	
Jan-Feb 2020	5.5	(3.8 - 8.0)	4.5	(2.8 - 7.1)	2.1	(1.2 - 3.6)	1.8	(0.9 - 3.4)	1.2	(0.7 - 2.1)	0.4	(0.1 - 1.2)	1.1	(0.5 - 2.4)	0.2	(0.0 - 1.0)	1198
March-Dec 2020	8.4	(7.2 - 9.9)	4.0	(3.1 - 5.2)	2.9	(2.3 - 3.7)	2.5	(1.8 - 3.5)	2.0	(1.4 - 3.0)	1.6	(1.0 - 2.6)	0.4	(0.2 - 0.9)	0.4	(0.2 - 0.8)	3260

Notes: Results shown are survey-weighted percentages, and 95% confidence intervals. MCPC = Mistreatment by Care Providers during Childbirth. MCPC Ignore = Health care clinicians ignored you, refused your request for help, or failed to respond to requests for help in a reasonable amount of time. MCPC Shout = Health care clinicians (doctors, midwives, or nurses) shouted at or scolded you. MCPC Other = Any other mistreatment. MCPC Force = Health care clinicians threatened to withhold treatment or forced you to accept treatment you did not want. MCPC Privacy = Your physical privacy was violated (e.g., being uncovered or having people in the delivery room without your consent). MCPC Info = Your private or personal information was shared without your consent. MCPC Threat = Health care clinicians threatened you in any other way. MCPC Abuse = You experienced physical abuse (including aggressive physical contact, inappropriate sexual conduct, refusal to provide anesthesia for an episiotomy, etc.). LGBTQ= Lesbian, Gay, Bisexual, Transgender, Queer; SWANA= Southwest Asian, Middle Eastern or North African; NHPI=Native Hawaiian or Pacific Islander; NAAN=Native American or Alaskan Native; SUD=Substance Use Disorderl; IPFV=Initimate Partner or Family Violence; PHE=Public Health Emergency.

© 2024 Liu C et al. *JAMA Network Open.*
