# Supplementary material for: Disparities in Mistreatment During Childbirth
Source: JAMA Netw Open. 2024 Apr 4;7(4):e244873. doi: 10.1001/jamanetworkopen.2024.4873 (PMC11192180; doi:10.1001/jamanetworkopen.2024.4873)
Supplement: Supplement 2. — Data Sharing Statement [file jamanetwopen-e244873-s002.pdf]

Data Sharing Statement

Liu. Disparities in Mistreatment During Childbirth. *JAMA Netw Open*. Published April 04, 2024.
doi:10.1001/jamanetworkopen.2024.4873

Data
Data available: No

Additional Information
Explanation for why data not available: We have individual data use agreements with each study jurisdiction that do not allow for researchers outside the DUA to have access to the data.
